# Development of a Consumer Health Vocabulary by Mining Health Forum Texts Based on Word Embedding: Semiautomatic Approach

**DOI:** 10.2196/12704

**Published:** 2019-05-23

**Authors:** Gen Gu, Xingting Zhang, Xingeng Zhu, Zhe Jian, Ken Chen, Dong Wen, Li Gao, Shaodian Zhang, Fei Wang, Handong Ma, Jianbo Lei

**Affiliations:** 1 Synyi Research Shanghai China; 2 Center for Medical Informatics Peking University Beijing China; 3 Harbin Medical University Harbin China; 4 School of Stomatology Peking University Beijing China; 5 APEX Data & Knowledge Management Lab Shanghai Jiao Tong University Shanghai China; 6 Department of Healthcare Policy and Research Weill Cornell Medicine New York, NY United States; 7 School of Medical Informatics and Engineering Southwest Medical University Luzhou city, Sichuan Province China

**Keywords:** consumer health vocabulary, word embedding, representation learning, natural language processing, consumer health information, ontology enrichment

## Abstract

**Background:**

The vocabulary gap between consumers and professionals in the medical domain hinders information seeking and communication. Consumer health vocabularies have been developed to aid such informatics applications. This purpose is best served if the vocabulary evolves with consumers’ language.

**Objective:**

Our objective is to develop a method for identifying and adding new terms to consumer health vocabularies, so that it can keep up with the constantly evolving medical knowledge and language use.

**Methods:**

In this paper, we propose a consumer health term–finding framework based on a distributed word vector space model. We first learned word vectors from a large-scale text corpus and then adopted a supervised method with existing consumer health vocabularies for learning vector representation of words, which can provide additional supervised fine tuning after unsupervised word embedding learning. With a fine-tuned word vector space, we identified pairs of professional terms and their consumer variants by their semantic distance in the vector space. A subsequent manual review of the extracted and labeled pairs of entities was conducted to validate the results generated by the proposed approach. The results were evaluated using mean reciprocal rank (MRR).

**Results:**

Manual evaluation showed that it is feasible to identify alternative medical concepts by using professional or consumer concepts as queries in the word vector space without fine tuning, but the results are more promising in the final fine-tuned word vector space. The MRR values indicated that on an average, a professional or consumer concept is about 14th closest to its counterpart in the word vector space without fine tuning, and the MRR in the final fine-tuned word vector space is 8. Furthermore, the results demonstrate that our method can collect abbreviations and common typos frequently used by consumers.

**Conclusions:**

By integrating a large amount of text information and existing consumer health vocabularies, our method outperformed several baseline ranking methods and is effective for generating a list of candidate terms for human review during consumer health vocabulary development.

## Introduction

### Background

In 2015, a survey of Chinese internet users showed that medicine and health care are the two most popular searched topics and accounted for 55.15% of all searches [1]. However, it is difficult for most users to express medical concepts using professional terms such as bronchus, brain, and extracellular space [2-4], and online forums and news media explain such professional medical terms with very little detail. The gap between consumer language and medical terminology makes searching and retrieving information difficult and biases the understanding of health information [5-7].

Development of the consumer health vocabularies, which map languages of consumers and medical experts, is a potential way to bridge the gap. Several commercial and noncommercial groups, such as Apelon and Public Health Terminology by Intelligent Medical Objects, and Open Access and Collaborative Consumer Health Vocabulary [8], tried to bind consumer health vocabularies with the Unified Medical Language System (UMLS) or the International Classification of Diseases (ICD). Several factors dominate the expansion of a quality consumer health vocabulary: a comprehensive search to identify related nonstandard expressions, abbreviations and common typos, a consensus between consumers’ point of view and professional classification, and periodic updating for new terms. These factors make the expansion process complicated, costly, and time-consuming.

To accelerate the expansion process, researchers developed many approaches to extract and map consumer terms automatically or semiautomatically, including the n–gram-based approach [9], pattern-based approach [7], co-occurrence analysis [10], and machine learning methods [9,11]. Although the consumer health vocabularies mined through these hand-crafted heuristic approaches are more accurate, many relevant pairs could be missing. Recent theoretical and experimental results from Wang et al [12] showed that matching professional-consumer concept pairs through text embedding approaches can capture the semantic similarities between professional concepts and consumer concepts, thus yielding a high recall. However, with only unsupervised algorithms, many irrelevant pairs could be generated. In order to retain the advantage of text embedding and improve precision as much as possible, we propose a semisupervised representation learning method to make the concept embedding specific in the consumer vocabulary mining process. With the knowledge introduced by the reviewer, concept embeddings can continuously improve themselves. Our approach provides a related consumer term list sorted by their semantic distance to a particular medical term and helps reviewers identify synonym pairs efficiently. We extracted consumer health terms from one of the most popular health forums in China and manually evaluated the performance of our approach. The experimental results are promising, showing performance improvement of up to 16% with a small amount of seed pairs.

### Synonym Identification

Two mainstream approaches for identifying synonyms are rule-based algorithm and word similarity measurement. A rule-based algorithm identifies synonyms by semantic patterns. For example, Vydiswaran et al took advantage of common linking phrases such as “also called,” “also known as,” and “also referred to as” to extract synonyms from Wikipedia [7]. There are many ways to calculate word similarities, including n-gram, edit-distance (Levenshtein distance), WordNet-distance, and cosine-distance between word vectors [13-16]. Among them, training distributed word vectors and extracting synonyms from top closest words are the most popular ways. Henriksson et al created word vectors using latent semantic analysis with random indexing and permutation to identify medical synonyms and abbreviation-expansion pairs [17,18]. He et al created word vectors including linguistic, contextual, and statistical features and used K-means to gather new consumer health terms on social media [19]. Elhadad et al created word vectors combining both contextual and semantic features and cluster terms from breast cancer forums into predefined semantic categories [20]. Wang et al created word vectors using word2vec, an open-source natural language processing tool released by Google, to extract symptoms from UMLS [12].

### Development of Consumer Health Vocabularies

As early as 1998, Marshall [21] mapped the consumer health terminology from WellMed (a health care website) to SNOMED (Systematized Nomenclature of Medicine) and UMLS, which helped patients search information using nonprofessional expressions. In 2001, Patrick expanded UMLS, the Eurodicautom of the European Commission’s Translation Service, and the European Commission Glossary of popular and technical medical terms, by adding words from the Dictionary of American Regional English, but only focused on diabetes-related terms [22]. Both Marshall and Patrick constructed their consumer health vocabularies manually, which is inefficient and unscalable. In 2005, Zeng developed a two-step approach, which combined corpus-based text analysis and manual review, to build an open-source consumer health vocabulary [23]. To reduce the labor in term mapping, Zeng improved the two-step approach by adding n-gram, logistic regression, and even natural language processing and machine learning algorithm (parts of speech, noun phrases, and named entities recognition) [9-11]. Since then, the two-step semiautomatic approach—term identification algorithms followed by manual review—evolved into a common practice in many consumer health vocabulary researches [7,10,24].

## Methods

### Overview

**Figure 1:**
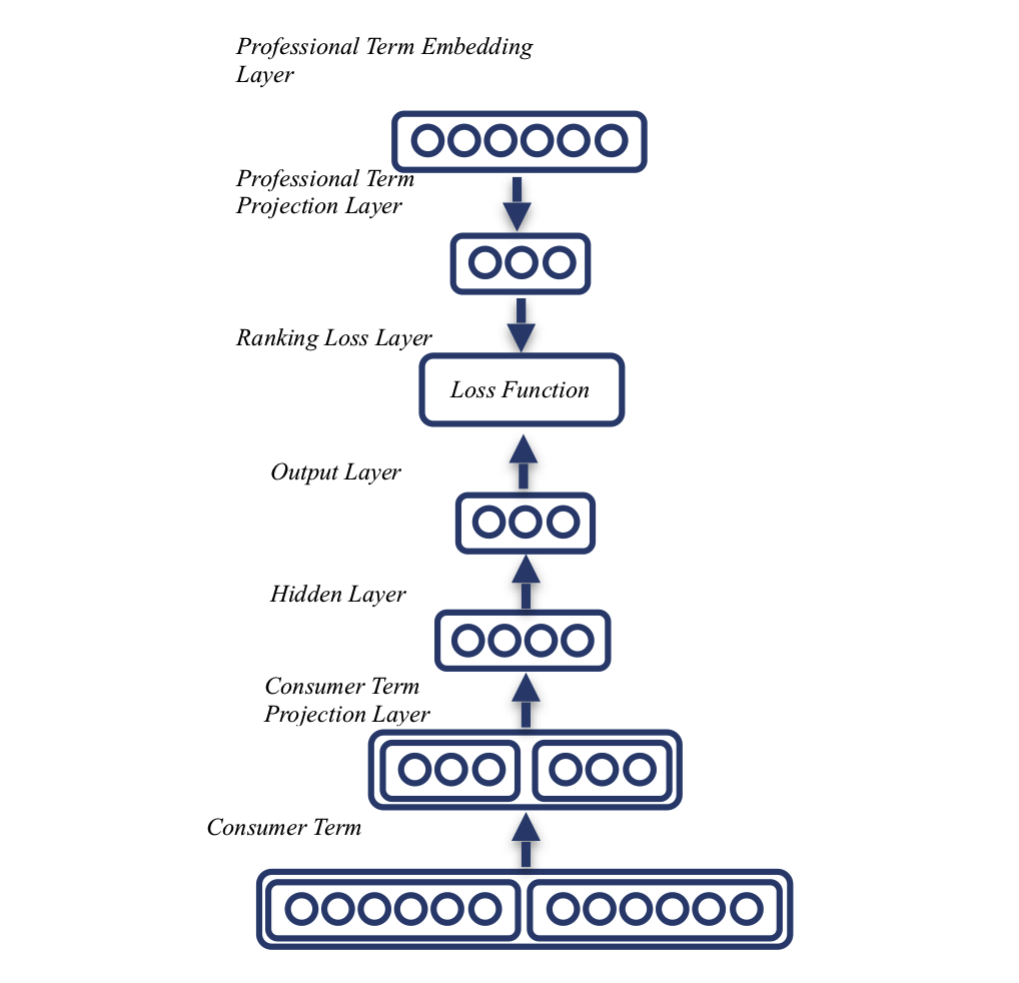
The overall architecture of our model. The consumer and professional terms start from both ends of the model, going through some embedding and projection layers, so that they are projected into a unified semantic space, where the ranking loss will be measured.

To alleviate the problems mentioned above, we propose a consumer health term–finding framework based on a distributed word vector space model. The overview of the framework is shown in Figure 1. The workflow can be interpreted as a feed-forward neural network. The first step of our approach requires a corpus of raw text for the unsupervised pretraining of the embedding matrix *E* as the embedding layer (Figure 1). Text embedding approaches have proven to be very effective in capturing the similarities between words and phrases, which can yield professional-consumer concept pairs that cannot be found by feature- and pattern-based methods.

We used THULAC (Tsinghua University - Lexical Analyzer for Chinese) [25], a Chinese segmentation tool, to change Chinese text into words. Thereafter, word embedding tools were used to compute the vector space, as described above. We collected professional concepts from the official Chinese version of ICD-10, calculated the frequency of words in the corpus, and extracted those with a count of over 1000 and their corresponding consumer concepts with context as seed pairs. All the weights were initialized uniformly at random. In order to obtain a model that takes professional concepts as input and consumer concept list as output after training, we introduced an embedding space–adapting process consisting of an embedding projection and a supervised ranking method. The embedding projection contains a projection layer, a hidden layer, and a target projection layer (Figure 1) to achieve a “smaller” embedding space that preserves more supervisory signal.

The output concept list is not necessarily the most similar word of the input concept in the word vector space, but with the supervised signal of seed terms, their similarity is more of a measure of professional-consumer concept pair similarity. A special ranking loss function is used in the ranking loss layer to calculate the similarity of professional-consumer concept pairs. After manually selecting the output professional-consumer concept pairs, we input the selection results into the training data. New professional-consumer concept pairs were discovered through iterations.

### Word Embedding

Word embeddings are generally trained to reconstruct linguistic contexts of words by optimizing an objective function that can be measured without annotations. One popular approach is to estimate the embeddings by maximizing the probability that the words within a given window size are predicted correctly. Word embedding takes a large corpus of text as its input and produces a vector space, with each unique word in the corpus assigned a corresponding vector in the space. In word embedding training, one of the key issues is the formulation of the training objective function, minimization or maximization of which may produce meaningful word vector representations. Ideally, the training objective function should reflect the fact that the semantic word similarities measured on learned word vectors are consistent with human cognition. Recently Wang et al [26] performed a comprehensive comparative study on the different word embedding techniques in biomedical texts. Of those, we chose three popular word embedding methods: Word2Vec [27], Global Vectors (GloVe) [28], and FastText [29].

#### Word2Vec

Word2Vec is a widely used method in natural language processing for generating word embeddings. It has two different training strategies: (1) Continuous Bag-of-Words, in which the model is given a sequence of words without the middle one and attempts to predict this omitted word, and (2) Skip-Gram, in which the model is given a word and attempts to predict its neighboring words. In both cases, the model consists of only a single weight matrix (apart from the word embedding), which results in a fast log-linear training process that can capture semantic information [26].

#### Global Vectors

The GloVe method was proposed by Pennington et al [28] and obtained state-of-the-art results for syntactic and semantic analogies tasks. This method has a co-occurrence matrix M that is constructed by looking at the context words. Each element *M*_ij_ in the matrix represents the probability of the word *i* being similar to the word *j*. In the matrix M, the vectors are randomly generated and trained with the equation *P* (*w*_i_, *w*_j_)=*log* (*M*_ij_)=*w*_i_*w*_j_+*b*_i_+*b*_j_, where *w*_i_ and *w*_j_ are word vectors and *b*_i_ and *b*_j_ are biases.

#### FastText

FastText is a recently developed method [29] proposed by the same group who developed word2vec, in which the embeddings are associated with character n-grams and the words are represented as the summation of these representations. Specifically, a word representation is induced by summing character n-gram vectors with vectors of surrounding words. Therefore, this method attempts to capture morphological information to induce word embedding.

### Adapting Embedding With Supervised Training

As mentioned in the Introduction, word embedding is a useful unsupervised technique to capture the similarity between words and phrases, which can yield high recall of professional-consumer concept pairs. It can also be used as a pretraining phase prior to supervised training. However, even if the embeddings provide compact, real, valued representations of each word in a vocabulary, it only indicates that word embeddings produce a semantic space that models synonymy to a certain degree. Current methods use pretrained embedding to initialize model parameters and then use the labeled data to guide them for the intended task (eg, we use professional-consumer concept pairs that already exist as the supervision to produce a semantic space dedicated to finding such pairs). If, as in our case, only a small amount of supervised data are available, this can lead to severe overfitting. Furthermore, rare words will receive very few updates and their embedding will be poorly adapted for our task. We propose two solutions to avoid these problems.

#### Embedding Projection

Let denote the original embedding matrix obtained. We define the adapted embedding matrix as the multiplication *S* • *E*, where the projection matrix and *s*< *e*. We estimate the parameters of the matrix *S* using the labeled dataset, while *E* is kept fixed. In other words, we determine the optimal projection of the embedding matrix *E* into a subspace. The ideal embedding subspace relies on two fundamental principles:

1. With dimensionality reduction of the embedding, the model can better fit the complexity of our consumer health vocabularies task or the amount of available data. As the number of professional-consumer concept pairs increase, the size of the embedding can be adjusted.
2. Using a projection, all embeddings are indirectly updated, not only for the words present in the labeled dataset.

Let *M*=[*w*_1_… *w*_n_] denote a message of n words. Each column w∈{0,1}^v^^×^^1^ of m represents a word in one-hot form. is the projection vector for each word, given by *P*= *S* • *E* • *M*. A simple adapting rule is to keep the original *S* fixed and append a new random initial matrix to *S* to obtain the new *S’* for retraining.

Compared to a conventional feed-forward network employing embedding for natural language, two main differences arise. First, the input layer is factorized into two components—the embedding attained in unsupervised form *E* and the projection matrix *S*. Second, the size of the subspace in which the embeddings are projected is much smaller than that of the original embedding with typical reductions above one order of magnitude. As is usual in this kind of model, all the parameters can be trained with gradient methods, using the back-propagation update rule.

#### Supervised Ranking Method

One of the challenges for supervised word embedding training is the difficulty of defining the exact similarity values between two words. Especially in our case, the professional concept and the consumer concept are different. The similarity measure is affected by many factors such as the dimensionality of the embedding, the employed learning algorithms, and the corpus size. Although the similarity values are quite different, the ranking of similarity values is more robust than the values itself.

Inspired by this finding, we employed ranking information as the supervised training targets. The ranking loss function 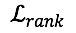
is obtained as,


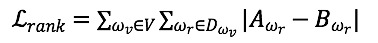


where *V* is the vocabulary, *ω*_v_ is a specific word, and 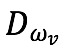
is the set of synonym words of *ω*_v_ in the labeled set. 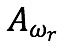
is the rank of *ω*_r_ in the labeled set, and 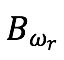
is the rank of *ω*_r_ according to its cosine similarity with *ω*_v_ measured in the embedding space.

Because the ranking loss is not differentiable, we choose to minimize the semantic similarity loss between the desired ranking position and the real ranking position in the embedding space as a surrogate. Given the desired ranking position, the similarity value corresponding to the desired ranking position is employed as the real training target. Minimizing the difference of similarity values between the desired position and the real position may also reduce the ranking loss. The similarity value lies in function 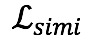, given below, where 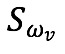
denotes the sorted similarity values for word *ω*_v_:


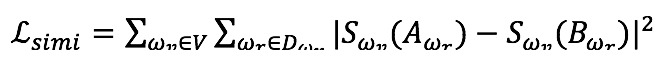


### Experiment

To evaluate the effectiveness of the proposed model, three groups of experiments were designed. The three kinds of word embeddings with different vector size were further trained by the proposed model and evaluated. The baselines were the original word embeddings described above. The effect of the projection layer was studied in the second group experiments. Two comparison groups were involved, one that used the standard structure without the projection layer and another that used the proposed projection layer.

### Data Sets

We tested our methods with the corpus obtained from two different Chinese communities to cover different perspectives. The Tianya community is one of the most popular online forums in China, and the data are open and easy to retrieve. The health care sector of the Tianya community—Tianya Hospital—has a large number of disease consulting posts initiated by consumers, and about 180 Mb of data are used in our experiment. Haodf is the largest Chinese medical question-and-answer website where all questions are created by patients and answered by doctors, and about 2 Gb of data are used in our experiment. We considered these to be ideal sources of consumer health corpus, used Scrapy [30] for a full-text crawling from those corpora, and removed user information before further processing. The messages from the consumer forums were preprocessed as follows: URLs were replaced with a token URL and words occurring less than 30 times in the corpus were replaced by a special UNKNOW symbol. We collected professional concepts from the Chinese official version of ICD-10, calculated the frequency of words in these two corpora, extracted them with a count of over 1000, and manually collected their corresponding consumer concepts with context as seed pairs. The annotation process was performed by one medical professional and reviewed by three medical professionals. We finally obtained 224 seed pairs that all three reviewers consistently agreed upon.

### Evaluation Method

We use the mean reciprocal rank to evaluate the quality of word embeddings. Mean reciprocal rank is a statistic measure for evaluating any process that produces a list of possible response to a sample of queries and orders them by probability of correctness. The mean reciprocal rank is the average of the reciprocal ranks of results for a sample of queries Q: 
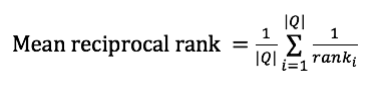
where *rank*_i_ refers to the rank position of the first relevant synonym for the *i*-th query.

We use a large collection of candidate medical concepts and build a small set of ground truth professional-consumer concept pairs. We randomly select 100 pairs from our seed pairs for evaluation.

## Results

### Principal Findings

In general, the performance of the proposed method is detailed in Table 1. All word embeddings are significantly enhanced after fine tuning. The performance of the best word embedding is FastText, with a 400-dimensional vector with Haodf and projection matrix size set to 40, and it is also significantly improved in all datasets. The rich n-gram features used in FastText are important in Chinese synonym finding and have much higher performance than others. These remarkable improvements demonstrate that our method may transfer the complementary knowledge from the weak embeddings into the strong embeddings.

### Effect of the Projection Layer

Table 2 shows the system performance with no projection matrix and different projection matrix size. As baselines, we considered a simple log-linear approach, which uses the unsupervised embeddings directly as features in a log-linear classifier. We tested the model performance. Furthermore, we observed that updating the embeddings always led to inferior results. This suggests that pretrained embeddings should be kept fixed, when little labeled data are available to retrain them.

### Manual Review of the Recommended Consumer Health Terms

In order to ensure the accuracy of professional-consumer concept pairs, manual review is inevitable. Table 3 showed the top 10 candidates for word “diarrhea” (“腹泻”) provided by our method for reviewers. Most words illustrated here are symptoms or clinical findings related to “diarrhea,” such as “vomiting” (“呕吐”), “abdominal pain” (“腹痛”), and “dyspepsia” (“消化不良”). We see two synonyms in the table: “having loose bowels” (“拉肚子”, ranked third) and “diarrhea” (“腹泄”, ranked seventh). The former is a consumer health term that is rarely used by professionals, and the latter is a typo of “diarrhea” (“腹泻”). In the manual review, researchers reviewed a sample of the candidate terms suggested by the system to assess whether these terms should be added into the consumer health vocabularies.

**Table 1:** Performance of three word embedding methods with different embedding sizes. Italicized values indicate the best performance of the date set.

Corpus and tuning state		GloVe^a^			Word2Vec			FastText		
		100^b^	200	400	100	200	400	100	200	400
**Tianya**										
	Before	0.266	0.263	0.282	0.289	0.296	0.272	0.341	0.340	0.319
	After	0.313	0.308	0.320	0.325	0.338	0.341	0.355	0.362	*0.371*
**Haodf**										
	Before	0.270	0.273	0.289	0.288	0.290	0.295	0.320	0.322	0.331
	After	0.321	0.326	0.332	0.313	0.344	0.346	0.361	0.365	*0.385*

^a^GloVe: Global Vectors.

^b^Values in this row indicate embedding size.

**Table 2:** Performance of FastText-200 with different sizes of the projection matrix.

Corpus	Projection matrix size				
	0^a^	20	40	80	160
Tianya	0.350	0.352	0.362	0.357	0.345
Haodf	0.338	0.342	0.365	0.360	0.331

^a^Projection matrix size 0 is used to denote the baseline (log-linear model).

**Table 3:** Top 10 candidates for the seed word “diarrhea.”

Rank	Medical words in Chinese	Medical words in English
1	呕吐	Vomiting
2	腹胀	Ventosity
3	拉肚子	Having loose bowels
4	腹痛	Abdominal pain
5	便秘	Constipation
6	消化不良	Dyspepsia
7	腹泄	Diarrhea
8	厌食	Anorexia
9	肠鸣	Borborygmus
10	返酸	Acid reflux

## Discussion

Bridging the language gap between consumers and medical professionals is a fundamental problem in medical internet research. There has been some research on building the consumer health vocabulary for English medical terms, but the research on other languages is scarce. The model developed in this paper was evaluated using Chinese terms and could help professionals collect consumer health vocabularies related to certain clinical topics and discover synonyms in a more effective and efficient way.

From the methodology perspective, we adopted unsupervised word embedding as the backbone of our approach. This mechanism encodes words into vectors based on the context they are likely to be put into and projects them into a common semantic space. We further fine-tuned the word embeddings to make them align with the limited supervision information provided. A previous study used word vectors trained on a large-scale corpus to explore semantic relationships such as analogy, subordination, and comparison [26,27]. In our corpus, the context of a diagnostic term could always be related diseases, symptoms, and drugs. Therefore, the embeddings of similar terms or synonyms with similar context will be close to each other in the space after the training process.

Our algorithm can correctly identify over 80% of the synonyms by just searching from the top 10 candidates of a certain medical term. We further summarize these synonyms into three classes: (1) Colloquial expressions; for example, consumers say “having loose bowels” (“拉肚子”) rather than “diarrhea” (“腹泻”) and “zits” (“青春痘”) rather than “acne” (“粉刺”). (2) Typos; for example, consumers always misspell “腹泻” (“diarrhea”) as “腹泄” and “黄疸” (“jaundice”) as “黄胆.” (3) The symptoms or findings from traditional Chinese medicine; for example, Chinese medicine refers to “stomachache” (“胃痛”) as “epigastric pain” (“胃脘痛”). Besides the “typo” synonyms, other two classes of synonyms do not necessarily share common characters with each other or source terms. Therefore, simple character-based matching approaches such as n-gram and edit-distance do not help in these cases. Semantic pattern-based algorithms depend less on exact common characters; however, consumers in online communities and social media express themselves in a more casual way, and we may not be able to create and maintain a comprehensive semantic pattern list to capture all the variations and diversities. Our method can fill in such a language gap and effectively expand the synonyms and consumer health vocabularies. We validated the effectiveness of our approach with the 180-Mb Tianya corpus and the 2-Gb Haodf corpus. The results indicate that the larger the corpus, the better the learning.

One limitation of our approach is that we cannot handle a case when a new professional term is needed in the vocabulary, especially the newly formulated professional term, for example, the extracellular space and the interstitial system [31]. This is because we adopted a matching-based framework. However, it is not difficult to extend the current algorithm to gain such capability. For example, we can normalize the similarity between a specific consumer term to all professional terms in the dictionary and thus make these similarities a probability distribution. Thereafter, we can use entropy for this distribution to determine whether we need a new professional term. A high entropy indicates that the consumer term is not really similar to any of the existing professional terms, and thus, a new term may be needed.

For the first time in the Chinese medical terminology field, this study verified the effectiveness of word semantic representations and their potential for linking narrative consumer terms to clinical terms. This approach can discover consumer expressions such as spelling errors and nonstandard abbreviations, which are usually missed in the traditional consumer health vocabularies, and enrich the consumer health vocabularies to meet consumer requirements for information retrieval. The candidate consumer term list automatically generated by our model can be employed as an important reference for professionals to discover synonyms in a more efficient way.

## Abbreviations

GloVe: Global Vectors
ICD: International Classification of Diseases
MRR: mean reciprocal rank
SNOMED: Systematized Nomenclature of Medicine
THULAC: Tsinghua University - Lexical Analyzer for Chinese
UMLS: Unified Medical Language System
